# RNA-Containing Immune Complexes Formed by Anti-Melanoma Differentiation Associated Gene 5 Autoantibody Are Potent Inducers of IFN-α

**DOI:** 10.3389/fimmu.2021.743704

**Published:** 2021-10-15

**Authors:** Kaiwen Wang, Jiangfeng Zhao, Wanlong Wu, Wenwen Xu, Shuhui Sun, Zhiwei Chen, Yakai Fu, Li Guo, Hui Du, Shuang Ye

**Affiliations:** Department of Rheumatology, Renji Hospital, Shanghai Jiao Tong University School of Medicine, Shanghai, China

**Keywords:** dermatomyositis, anti-MDA5 autoantibody, RNA-containing immune complexes, interferon pathway, IFN-α

## Abstract

**Objective:**

Anti-melanoma differentiation-associated gene 5 (MDA5) autoantibody is a distinctive serology hallmark of dermatomyositis (DM). As an autoantigen, MDA5 is a cytoplasmic RNA recognition receptor. The aim of this study was to address the question of whether the RNA-containing immune complex (IC) formed by MDA5 and anti-MDA5 could activate type I interferon (IFN) response.

**Method:**

Patients with anti-MDA5^+^ DM (*n* = 217), anti-MDA5^−^ DM (*n* = 68), anti-synthase syndrome (ASyS, *n* = 57), systemic lupus erythematosus (SLE, *n* = 245), rheumatoid arthritis (RA, *n* = 89), and systemic sclerosis (SSc, *n* = 30) and healthy donors (HD, *n* = 94) were enrolled in our studies. Anti-MDA5 antibody was detected by line blotting, enzyme-linked immunosorbent assay (ELISA), immunoprecipitation, and Western blotting. Cytokine profiling was determined by multiplex flow cytometry, and IFN-α was further measured by ELISA. Type I IFN-inducible genes were detected by quantitative PCR (qPCR). RNA–IC binding was analyzed by RNA immunoprecipitation. Plasmacytoid dendritic cells (pDCs) derived from healthy donors were cultivated and stimulated with MDA5 ICs with or without RNase and Toll-like receptor 7 (TLR-7) agonist. The interaction between MDA5 ICs and TLR7 was evaluated by immunoprecipitation and confocal microscopy.

**Results:**

According to our in-house ELISA, the presence of anti-MDA5 antibody in 76.1% of DM patients, along with 14.3% of SLE patients who had a lower titer yet positive anti-MDA5 antibody, was related to the high level of peripheral IFN-α. ICs formed by MDA5 and anti-MDA5 were potent inducers of IFN-α *via* TLR-7 in an RNA-dependent manner *in vitro*.

**Conclusion:**

Our data provided evidence of the mechanistic relevance between the anti-MDA5 antibody and type I IFN pathway.

## Introduction

Anti-melanoma differentiation-associated gene 5 (MDA5) antibody is considered a specific serological marker in dermatomyositis (DM), which is typically linked to the amyopathic phenotype and rapidly progressive interstitial lung disease (ILD) (1). The pathogenesis of anti-MDA5 antibody-positive (anti-MDA5**^+^**) DM is largely unknown; however, possible genetic factors (with East Asian predisposition) and environmental triggers (such as viral infection) have been implicated (2). MDA5, encoded by the *IFIH1* gene, is the key sensor of viral double-strand RNA (dsRNA) and belongs to the retinoic acid-induced gene I (RIG-I)-like receptors (3), which are actively involved in the type I interferon (IFN-α and IFN-β) pathway. Indeed, type I IFN is well conceived to play a crucial role in the pathogenesis of DM and has profound treatment implications (4). It is noteworthy that systemic lupus erythematosus (SLE) is also characterized by the aberrant activation of the type I IFN pathway (5). There is strong genetic evidence for a link between cytoplasmic RNA sensing pathways (RIG-I/MDA5) and SLE (6, 7). The current knowledge suggested that the anti-MDA5 antibody, as a serological marker, is more likely to be a by-product of an “overloaded” type I IFN pathway in DM (8–11). In the present study, however, we are asking the question whether the anti-MDA5 antibody could enhance the type I IFN response.

## Methods

### Patients

All DM patients fulfilled the 239th European Neuromuscular Centre (ENMC) criteria (12) with the presence of one of the five DM-specific autoantibodies (anti-MDA5, anti-TIF1γ, anti-SAE1, anti-NXP2, or anti-Mi2), which were determined by EUROLINE Autoimmune Inflammatory Myopathies 16 Ag assays (EUROIMMUN, Lübeck, Germany). Anti-synthase syndrome (ASyS) (12), rheumatoid arthritis (RA), systemic sclerosis (SSc), and SLE patients served as the disease controls, abiding by the respective classification criteria. A Bio-Plex Pro 2200 Luminex LiquiChip immunoassay (Bio-Rad, Hercules, CA, USA) was used to detect autoantibodies against extractable nuclear antigens (ENAs) for all patients. Healthy donor and patient samples were obtained with informed consent. The study protocol was approved by the Ethics Committee of Renji Hospital, Shanghai Jiao Tong University School of Medicine (ethics no. IRB# 2017-041). All participants provided written informed consent.

### ELISA for Anti-MDA5 Antibody

ELISA plates were coated overnight at 4°C with 10 μg/well of recombinant full-length MDA5 protein (Shanghai Free Zone Biotechnology Co., Ltd., Shanghai, China). After blocking with 1% bovine serum albumin (BSA), patient sera were used at 1:101 dilution and horseradish peroxidase-labeled anti-human immunoglobulin G (IgG) antibodies (1:60,000; Jackson Laboratory, Bar Harbor, ME, USA) were incubated for 30 min, then stopped by tetramethylbenzidine reagent (Invitrogen, Waltham, MA, USA) and the absorbance read at 450 nm (Sprinter XL, EUROIMMUN, Lübeck, Germany). The cutoff value for this in-house anti-MDA5 antibody ELISA was set as the mean + 3SD of 94 healthy donors. To evaluate the consistency between this in-house and a commercial anti-MDA5 antibody ELISA (MBL, Tokyo, Japan) assay, 92 subjects (DM = 47, HC = 38, and SLE = 7) were tested back to back, with good agreement (*κ* = 0.847, *p* < 0.001).

### Cytokine Assays

Serum cytokine levels [including IFN-α, IFN-β, IFN-γ, IL-1β, interleukin 2 (IL-2), IL-4, IL-5, IL-6, IL-8, IL-10, IL-12p70, IL-17A, IL-17F, IL-22, tumor necrosis factor alpha (TNF-α), TNF-β, and granulocyte–macrophage colony-stimulating factor (GM-CSF)] were measured with an AimPlex flow cytometer (Tianjin Kuang Bo Tongsheng Biotechnology Co., Ltd., Tianjin, China) and a commercial IFN-α ELISA kit (PBL Biomedical, Piscataway, NJ, USA) following the manufacturers’ protocols.

### Immunoprecipitation and Western Blotting

Immunoprecipitation (IP) assay using a cell lysate overexpressing Flag-tagged MDA5 was generated using a 293 cell line (Shanghai Free Zone Biotechnology Co., Ltd., Shanghai, China). Of the cell lysate, 100 μg was pre-cleared with protein A beads in NP40 lysis buffer (Thermo Fisher, Waltham, MA, USA) and immunoprecipitated with 5 µl patient serum. The immunoprecipitates were blotted with an anti-Flag antibody (Invitrogen, Waltham, MA, USA) and visualized using enhanced chemiluminescence (Thermo Fisher, Waltham, MA, USA) in a FluorChem M chemiluminescence imager (ProteinSimple, San Jose, CA, USA). Recombinant MDA5 protein (60 μg/lane) was electrophoresed on SDS-PAGE gels and transferred into nitrocellulose membranes for the immunoblotting assays. Sera were used at 1:201 dilution for MDA5 immunoblot.

### PBMC Isolation and SYBR Green qRT-PCR

Peripheral blood mononuclear cells (PBMCs) were prepared using Ficoll-Hypaque (TBD, Tianjin, China) density gradient centrifugation from DM and SLE patients. The total RNA extracted from PBMCs using the TRIzol reagent (Invitrogen, Waltham, MA, USA) was reverse transcribed into cDNA with the Superscript II Reverse Transcriptase Kit (Takara, Shiga, Japan). The transcription levels of type I IFN-inducible genes (*LY6E*, *OAS1*, *Mx-1*, *IFIT1*, and *IFIT3*) were measured using SYBR^®^ Premix Ex Taq™ II (Takara, Shiga, Japan) *via* an ABI Prism 7900 system. The primer sequences we have previously reported (13) are as follows: *LY6E*: 5′-CTTACGGTCCAACATCAGAC-3′, 5′-GCACACATCCCTACTGACAC-3′; *OAS-1*: 5′-GAAGGCAGCTCACGAAAC-3′, 5′-TTCTTAAAGCATGGGTAATTC-3′; *Mx-1*: 5′-GGGTAGCCACTGGACTGA-3′, 5′-AGGTGGAGCGATTCTGAG-3′; *IFIT1*: 5′-TCAAAGTCAGCAGCCAGTCTCA-3′, 5′-GCCTCCTTGGGTTCGTCTATAA-3′; *IFIT3*: 5′-AACTACGCCTGGGTCTACTATCACTT-3′, 5′-GCCCTTTCATTTCTTCCACAC-3′; and *GAPDH*: 5′-ATTGCCCTCAACGACCACTTTG-3′, 5′-TTGATGGTACATGAAAGGTGAGG-3′. The IFN score was calculated as the sum of the five normalized expression levels of the corresponding tar (14):


$$\text{IFN score}=\Sigma \frac{\text{Gen}{\text{e}}_{\text{DM or SLE}}-\bar{X}(\text{Gen}{\text{e}}_{\text{HD}})}{\text{SD}(\text{Gen}{\text{e}}_{\text{HD}})}$$

Gene_(DM or SLE)_ represents the relative expression of a specific gene in DM or SLE patients. The average value $\left(\bar{X}\right)$ and the standard deviation (SD) of the expression level of each target gene in healthy donors (HD, *n* = 10) were also presented.

### Serum IgG Purification and Immune Complex Formation

IgG from DM and SLE patient sera were purified using a HiTrap Protein G HP column (GE Healthcare, Chicago, IL, USA) and quantified using a BCA Protein Assay Kit (Sigma-Aldrich, St. Louis, MO, USA). The cytosolic protein and nuclear material of 293 cells (2 × 10^6^) overexpressing Flag-tagged MDA5 were extracted with the Paris ™ Kit (Invitrogen, Waltham, MA, USA) and the cytosolic protein and RNA concentrations titrated to 2 mg/ml and 400 ng/µl, respectively. One hundred microliters of cytosolic protein, 50 µl purified serum IgG at a concentration of 1 mg/ml, and 10 µL RNA formed the immune complex (IC).

### Purification and Activation of pDCs

Healthy donor blood buffy coat was obtained from Shanghai Blood Transfusion Center and fractionated over Ficoll gradients. Plasmacytoid dendritic cells (pDCs) were isolated from PBMCs using the Plasmacytoid Dendritic Cell Isolation Kit II (Miltenyi Biotec, Bergisch Gladbach, Germany). pDCs were cultured at a density of 3 × 10^4^ cells/well in 96-well plates in 200 µl complete RPMI supplemented with 10% fetal calf serum (FCS) and stimulated overnight before the levels of IFN-α and CD80 were measured. The ICs, TLR-7 agonist R848 (2 μg/ml), and RNase (5 μg/ml) were added (both from MedChemExpress, Princeton, NJ, USA) when indicated.

### RNA-Binding Protein Immunoprecipitation

ICs were lysed with the EZ-Magna RIP™ RNA-Binding Protein Immunoprecipitation Kit (Merck Millipore, Darmstadt, Germany) and incubated with goat anti-human IgG antibody-labeled protein A/G at 4°C overnight. The magnetic beads were washed six times with pre-cooled RIPA buffer. Immunoprecipitates were detected by Western blotting using antibody against MDA5 (1:1,000; Abcam, Cambridge, MA, USA). The purified RNA from immunoprecipitates was measured with 1.5% agarose gel electrophoresis.

### Co-Immunoprecipitation Assay

ICs and pDCs were incubated overnight. Thereafter, the pDCs were lysed with RIPA lysis buffer (Thermo Fisher, Waltham, MA, USA) on ice for 4 h. The cell lysates were centrifuged at 13,000 rpm for 15 min. The supernatants were subsequently collected and pre-incubated with ImmunoPure Immobilized Protein A (Pierce, Rockford, IL, USA) with gentle shaking at 4°C for 1 h. Subsequently, the mixture was centrifuged at 13,000 rpm for 15 min and the supernatants (1 mg protein) incubated with 1 µg of goat monoclonal anti-human IgG antibody for 1 h, after which they were incubated with 10 µl of protein A agarose beads with gentle agitation at 4°C overnight. After centrifugation, the pellets were washed with lysis buffer. The target proteins were detected by Western blotting using antibodies against MDA5 (1:1,000) and Toll-like receptor 7 (TLR7) (1:2,000; both from Abcam, Cambridge, MA, USA).

### Confocal Microscopy

For lysosome co-localization, pDCs were incubated overnight at 37°C with ICs (Alexa Fluor 488-conjugated serum IgG), cytosolic protein lysate, and nuclear lysate. Then, pDCs were allowed to adhere to poly-l-lysine-coated coverslips for 15 min, fixed with 1% paraformaldehyde in phosphate-buffered saline (PBS), and permeabilized with 0.25% saponin. The samples were stained with Alexa Fluor 647-conjugated anti-LAMP-1 in 0.25% saponin. They were then imaged using a Zeiss LSM510 META confocal microscope and the images analyzed for co-localization using SlideBook software.

### Statistics

Student’s *t*-tests, chi-square test, and Spearman’s correlation analysis were used as indicated. The clinical variables were only presented without imputation for those with missing data less than 10%. The in-house and commercial anti-MDA5 antibody detection ELISA kits used the kappa test for consistency comparison (15). All statistics were performed using GraphPad Prism 6.0, with *p* < 0.05 considered as statistically significant.

## Results

**Figure 1:**
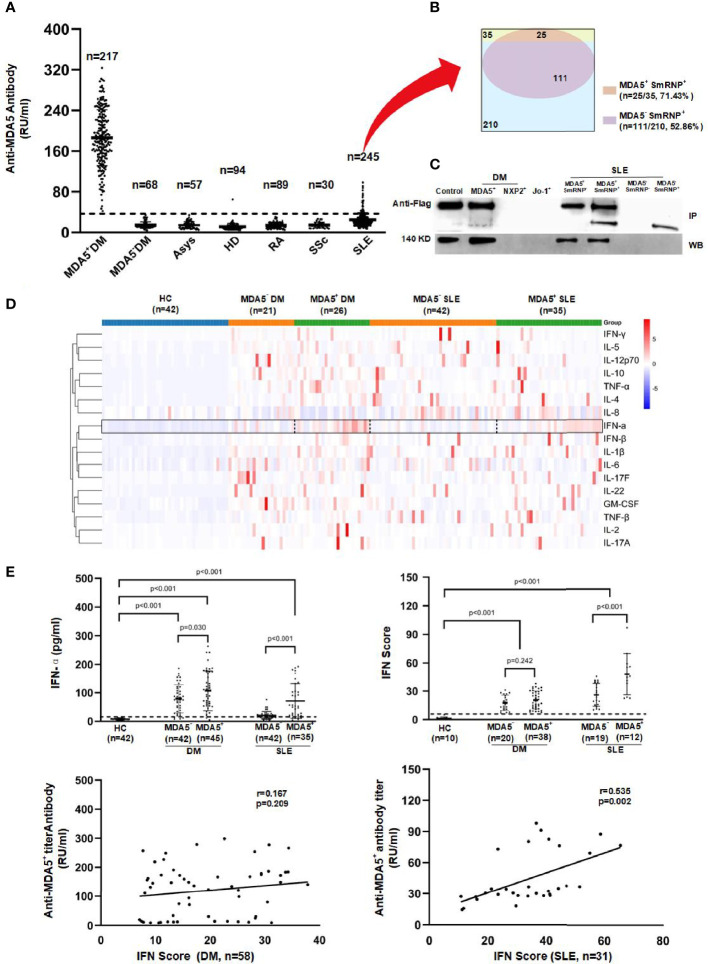
Anti-melanoma differentiation-associated gene 5 (MDA5) autoantibody and cytokine profiling in dermatomyositis (DM) and systemic lupus erythematosus (SLE) patients. **(A)** Quantitative anti-MDA5 measured by ELISA in anti-MDA5^+^ DM, MDA5^−^ DM (*n* = 68, anti-Mi-2α/β = 13, anti-NXP2 = 17, anti-SAE1 = 15, anti-TIF1γ = 23), anti-synthase syndrome (ASyS; *n* = 57, anti-Jo-1 = 10, anti-PL-7 = 12, anti-PL-12 = 12, anti-EJ = 10, anti-OJ = 13), SLE, systemic sclerosis (SSc), and rheumatoid arthritis (RA) patients and in healthy donors. The *dotted line* represents the cutoff value (35 RU/ml) of the anti-MDA5 antibody titer. **(B)** Overlaps among the anti-MDA5 and anti-SmRNP antibodies in SLE patients. **(C)** Immunoprecipitation (IP, *upper*) and Western blotting (WB, *lower*) were performed to validate the anti-MDA5 ELISA results (representative of more than five independent experiments). **(D)** Heat map of the cytokine expression profiling of DM and SLE patients. **(E)** Serum interferon alpha (IFN-α) and peripheral blood mononuclear cell (PBMC) IFN-inducible genes were measured by ELISA and SYBR green qRT-PCR, respectively. The *dotted line* represents the cutoff value (mean + 3SD of the values of healthy donors) of the serum IFN-α and IFN scores.

The clinical characteristics of the patients with DM and SLE are shown in **Table 1** (details of this anti-MDA5**^+^** DM patient cohort had been reported previously (16) and data for other disease controls including ASyS, RA, and SSc are in **Table 2**). The titers of the anti-MDA5 antibody according to ELISA for each group are presented in **Figure 1A**. The cutoff value of our in-house ELISA was set at 35 RU/ml, although this study did not include DM-specific antibody-negative DM patients. According to the results of our in-house ELISA, 76.1% of DM patients had anti-MDA5. Anti-MDA5 antibodies were at high titers among line blotting-established anti-MDA5^+^ DM patients, whereas they were consistently negative across the board among the disease controls, except for SLE. Of the SLE patients, 14.29% (35/245) had low-grade but positive anti-MDA5. Interestingly, anti-MDA5**^+^** SLE was associated with malar rash (with no DM features) and the presence of anti-SmRNP antibody (**Table 1** and **Figure 1B**). This in-house anti-MDA5 ELISA methodology was validated by Flag-tagged MDA5 immunoprecipitation and recombinant MDA5-based Western blotting (**Figure 1C**).

**Table 1:** Clinical and serological characteristics among patients with anti-MDA5^+/−^ dermatomyositis (DM) and systemic lupus erythematosus (SLE).

Index	DM			SLE		
	MDA5^−^ (*N* = 68)	MDA5^+^ (*N* = 217)	*p*-value	MDA5^−^ (*N* = 210)	MDA5^+^ (*N* = 35)	*p*-value
Sex, female, *n* (%)	28 (41.18)	84 (38.71)	0.776	192 (91.43)	33 (94.29)	0.747
Age (years), mean (SD)	54.39 (13.14)	49.91 (11.35)	**0.007**	36.05 (11.74)	35.23 (7.00)	0.687
Heliotrope/Gottron’s, *n* (%)	61 (89.71)	189 (87.10)	0.675	–	–	–
Malar rash, *n* (%)	–	–	–	43 (20.48)	13 (37.14)	**0.048**
Myositis, *n* (%)	52 (76.47)	27 (12.44)	**<0.001**	4 (1.90)	2 (5.71)	0.206
ILD, *n* (%)	11 (16.18)	217 (100.00)	**<0.001**	14 (6.67)	3 (8.57)	0.718
Fever, *n* (%)	7 (10.29)	139 (64.06)	**<0.001**	11 (5.24)	0 (0.00)	0.373
Arthralgia, *n* (%)	36 (52.94)	97 (44.70)	0.266	71 (33.81)	8 (22.86)	0.243
LN, *n* (%)	–	–	–	55 (26.19)	12 (34.29)	0.314
NPSLE, *n* (%)	–	–	–	5 (2.38)	1 (2.86)	>0.999
WBC (10^3^/µl), mean (SD)	7.46 (2.54)	6.92 (3.05)	0.220	5.77 (2.67)	5.29 (1,98)	0.317
HB (g/L), mean (SD)	127.89 (26.29)	124.54 (16.80)	0.244	117.40 (31.10)	120.00 (41.26)	0.665
Lymphopenia (<0.8 × 10^3^/µl), *n* (%)	11 (16.18)	128 (58.99)	**<0.001**	55 (26.19)	14 (40.00)	0.106
PLT (10^3^/µl), mean (SD)	209.05 (68.76)	196.67 (81.51)	0.298	201.22 (82.82)	201.42 (57.07)	0.453
CK_max_ (U/L), mean (SD)	2450.19 (5349.61)	279.33 (575.59)	**<0.001**	94.23 (60.32)	87.26 (74.21)	0.546
Ferritin (ng/ml), mean (SD)	841.45 (1032.73)	1677.12 (2434.33)	**0.032**	157.43 (177.09)	115.83 (123.12)	0.157
C3 (g/L), mean (SD)	0.95 (0.42)	1.01 (0.22)	0.200	0.83 (0.26)	0.88 (0.21)	0.107
C4 (g/L), mean (SD)	0.25 (0.10)	0.26 (0.08)	0.491	0.19 (0.10)	0.12 (0.06)	0.209
CRP (mg/L), mean (SD)	10.87 (24.36)	11.26 (14.97)	0.891	6.48 (12.86)	4.08 (8.27)	0.287
ESR (mm/h), mean (SD)	28.76 (22.02)	33.24 (20.74)	0.128	21.39 (22.54)	23.06 (17.49)	0.681
IgG (g/L), mean (SD)	14.08 (4.94)	15.29 (5.50)	0.214	14.38 (5.06)	17.17 (5.47)	**0.001**
Anti-dsDNA (IU/ml), *n* (%)	0 (0.00)	0 (0.00)	NA	120 (57.14)	19 (54.29)	0.854
Anti-Sm (AI), *n* (%)	0 (0.00)	0 (0.00)	NA	82 (39.05)	15 (42.86)	0.711
Anti-SmRNP (AI), *n* (%)	0 (0.00)	0 (0.00)	NA	111 (52.86)	25 (71.43)	**0.045**
Anti-Ribo P (AI), *n* (%)	0 (0.00)	0 (0.00)	NA	65 (30.95)	12 (34.29)	0.697
Anti-SS-A52 (AI), *n* (%)	3 (4.41)	139 (64.06)	**<0.001**	83 (39.52)	13 (37.14)	0.853
Anti-SS-A60 (AI), *n* (%)	0.00 (0.00)	0.00 (0.00)	NA	133 (63.33)	23 (65.71)	0.851
Anti-MDA5 titer (RU/ml), mean (SD)	**14.23 (6.37)**	**186.22 (54.98)**	**<0.001**	**20.68 (6.47)**	**51.68 (17.91)**	**<0.001**

Results are depicted as the mean and SD. Student’s t-test was used for continuous variables and Fisher’s exact test for dichotomous variables. MDA5^−^ DM included patients with anti-Mi-2α/β (n = 13), anti-NXP2 (n = 17), anti-SAE1 (n = 15), and anti-TIF1γ (n = 23).

ILD, interstitial lung disease; CK_max_, peak plasma CK; LN, lupus nephritis; NPSLE, neuropsychiatric systemic lupus erythematosus; WBC, white blood cell; HB, hemoglobin; PLT, platelet; CRP, C-reactive protein; ESR, erythrocyte sedimentation rate; IgG, immunoglobulin G. P-value <0.05 is statistically significant in bold text.

**Table 2:** Clinical and serological characteristics of patients with MDA5^−^ dermatomyositis (DM) and others.

Index	NXP2^+^ (*N* = 17)	TIF1γ^+^ (*N* = 23)	Mi-2α/β^+^ (*N* = 13)	SAE1^+^ (*N* = 15)	ASyS (*N* = 57)	RA (*N* = 89)	SSc (*N* = 30)
Sex, female, *n* (%)	11 (64.71)	10 (43.48)	12 (92.31)	14 (93.33)	35 (61.40)	70 (78.65)	21 (70.00)
Age (years), mean (SD)	48.59 (18.17)	57.00 (9.50)	53.77 (13.65)	58.00 (8.20)	53.03 (13.28)	57.81 (11.20)	52.10 (12.10)
Myositis, *n* (%)	14 (82.35)	17 (73.91)	13 (100.00)	8 (53.33)	22 (38.60)	0 (0.00)	0 (0.00)
ILD, *n* (%)	3 (17.65)	1 (4.35)	2 (15.38)	5 (33.33)	36 (63.16)	11 (12.36)	19 (63.33)
Fever, *n* (%)	4 (23.53)	3 (13.04)	0 (0.00)	0 (0.00)	16 (28.07)	4 (4.49)	0 (0.00)
Arthralgia, *n* (%)	11 (64.71)	13 (56.52)	8 (61.54)	4 (26.67)	14 (24.56)	55 (61.80)	2 (6.67)
WBC (10^3^/µl), mean (SD)	9.89 (4.94)	8.82 (3.59)	8.83 (2.75)	7.54 (1.79)	9.83 (4.04)	7.18 (3.01)	6.42 (2.70)
HB (g/L). mean (SD)	111.5 (24.82)	152.52 (28.46)	120.00 (6.97)	129.08 (17.65)	136.23 (12.60)	115.42 (24.18)	137.79 (30.38)
Lymphopenia (<0.8 × 10^3^/µl), *n* (%)	4 (23.53)	2 (8.70)	2 (15.38)	4 (26.67)	11 (19.30)	3 (3.37)	1 (3.33)
PLT (10^3^/µl), mean (SD)	212.07 (62.60)	244.04 (66.25)	265.00 (64.49)	237.25 (72.78)	242.33 (35.02)	296.57 (124.38)	253.33(77.59)
CKmax (U/L), mean (SD)	5,297.00 (8464.68)	1,312.77 (2479.27)	1,845.17 (1826.54)	306.30 (425.30)	1,164.82 (1904.91)	43.72 (46.63)	53.00 (37.22)
Ferritin (ng/ml), mean (SD)	1,616.22 (1614.39)	558.94 (578.12)	904.54 (829.75)	439.30 (549.87)	487.66 (514.54)	218.65 (243.52)	103.69 (91.17)
CRP (mg/L), mean (SD)	19.22 (38.74)	7.84 (11.74)	12.72 (21.38)	2.82 (0.68)	14.87 (19.03)	19.30 (28.87)	5.59 (7.49)
ESR (mm/h), mean (SD)	20.79 (22.75)	14.89 (13.36)	24.71 (13.47)	13.00 (10.55)	23.85 (19.69)	38.83 (27.67)	15.08 (11.75)
IgG (g/L), mean (SD)	10.55 (3.28)	15.90 (3.72)	15.33 (8.01)	14.00 (6.06)	14.47 (5.83)	22.85 (55.28)	16.36 (3.75)
Anti-SS-A52 (AI), *n* (%)	0 (0.00)	1 (4.35)	0 (0.00)	2 (13.33)	23 (40.35)	15 (16.85)	0 (0.00)
Anti-MDA5 titer (RU/ml), mean (SD)	**18.90 (8.64)**	**13.56 (5.87)**	**12.10 (4.21)**	**11.83 (1.31)**	**16.28 (5.58)**	**13.97 (6.14)**	**14.50 (5.79)**

Anti-MDA5, anti-melanoma differentiation-associated gene 5; ASyS, anti-synthase syndrome; RA, rheumatoid arthritis; ILD, interstitial lung disease; WBC, white blood cell; HB, hemoglobin; PLT, platelet; CRP, C-reactive protein; ESR, erythrocyte sedimentation rate; IgG, immunoglobulin G. P-value <0.05 is statistically significant in bold text.

Subsequently, we carried out cytokine profiling (including IFN-α, IFN-β, IFN-γ, IL-1β, IL-2, IL-4, IL-5, IL-6, IL-8, IL-10, IL-12p70, IL-17A, IL-17F, IL-22, TNF-α, TNF-β, and GM-CSF) in sera of DM and SLE patients with or without anti-MDA5. We found that IFN-α was significantly higher in anti-MDA5^+^ DM or SLE patients compared to their counterparts (**Figure 1D**). This was independently confirmed by an IFN-α ELISA. Furthermore, the IFN signature genes (*LY6E*, *OAS1*, *Mx-1*, *IFIT1*, and *IFIT3*) were measured and the IFN scores calculated; there was a positive correlation between the anti-MDA5 titer and the IFN scores among SLE patients (*p* = 0.002). However, this was not the case among DM patients (**Figure 1E**).

**Figure 2:**
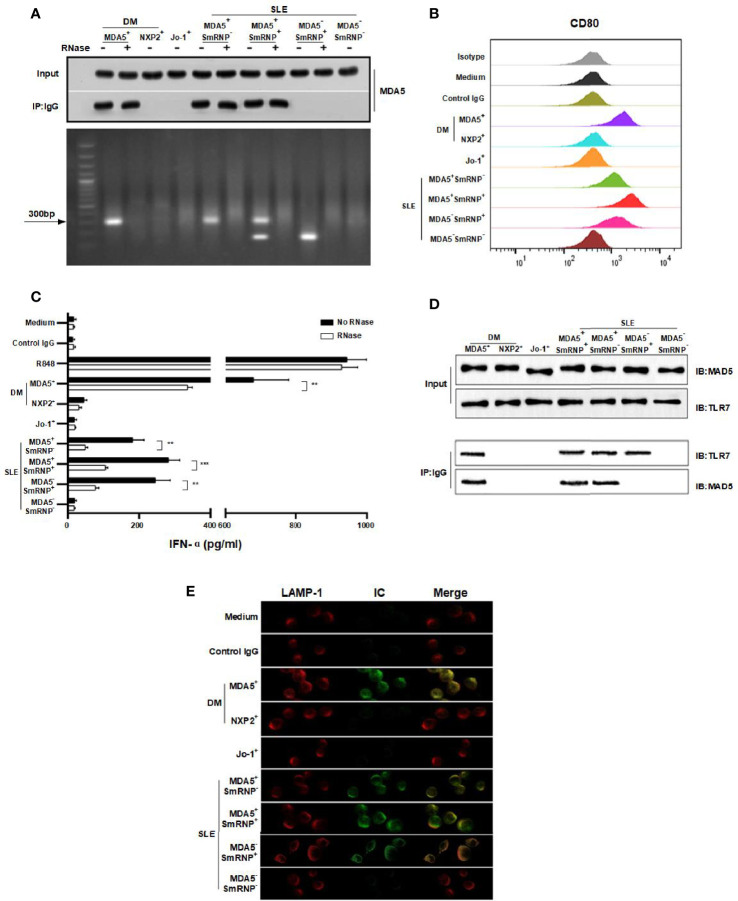
Anti-melanoma differentiation-associated gene 5 (MDA5) immune complexes (ICs) induce interferon alpha (IFN-α) production in plasmacytoid dendritic cells (pDCs). **(A)** MDA5 ICs contain RNA (~300 bp). **(B)** Purified pDCs were cultured with MDA5 ICs overnight and stained for CD80 expression. **(C)** RNA is required for MDA5 ICs to activate pDCs to release IFN-α. **(D)** Co-immunoprecipitation (co-IP) verified protein interaction between Toll-like receptor 7 (TLR7) and MDA5 ICs. **(E)** Confocal images show lysosome-associated membrane protein 1 (LAMP-1) (*red*), ICs (*green*), and the merged image (*yellow*). Results represent at least three biological independent repeats for all experiments **(A–E)**.

Based on the link of anti-MDA5 and IFN-α, we speculated that anti-MDA5 might work just like anti-ribonucleoprotein (anti-RNP), which is a typical IFN-α inducer in the form of RNA-containing ICs (17). We then tested this hypothesis using MDA5 and anti-MDA5 ICs to stimulate pDCs. Anti-RNP ICs were treated as the positive control. Indeed, RNA-containing anti-MDA5 ICs could activate pDCs, as evidenced by the upregulation of CD80 expression (**Figures 2A, B**). The IFN-α production of pDCs was enhanced by the stimulation of RNA-containing ICs, and the effect was more pronounced for the anti-MDA5**^+^** DM-derived ICs. This IFN-α inducer effect apparently required RNA since, in the presence of RNase, the IC-stimulated IFN-α production was significantly inhibited (**Figure 2C**). Further co-immunoprecipitation (co-IP) analysis confirmed the direct interaction between anti-MDA5 ICs and TLR7 (**Figure 2D**) and the indirect co-localization of ICs with TLR7 in lysosome (LAMP-1) in pDCs (**Figure 2E**) (18).

## Discussion

Anti-MDA5**^+^** DM is a life-threatening disease when complicated with rapidly progressive ILD. The anti-MDA5 antibody was considered to be pathognomonic, with little understanding in terms of its relevance to the pathogenesis. In the current study, using our in-house ELISA, we first identified that a small proportion of SLE patients also had autoantibody against MDA5, albeit with lower titer and probably lower affinity compared to that of anti-MDA5^+^ DM patients. It is intriguing that the presence of anti-MDA5 in SLE was not linked to DM features such as myositis or ILD; instead, typical lupus malar rash was more frequently documented. More importantly, these patients had salient peripheral IFN signature. In addition, the presence of anti-MDA5 in SLE was correlated with anti-RNP. It has been well established that SmRNP and anti-SmRNP IgG complexes could induce IFN-α production in pDCs through the TLR7 pathway (17). Thus, we came up with the hypothesis that MDA5, as an RNA sensor, could also exert an IC-mediated IFN-α stimulatory effect through pattern recognition receptors. Subsequently, anti-MDA5**^+^** DM patients, along with SLE patients presenting with anti-MDA5 or anti-RNP alone as controls, were subjected to a series of *in vitro* experiments in order to explore the possible antibody-mediated innate immune response.

We confirmed that anti-MDA5**^+^** DM, just as in anti-MDA5**^+^** SLE, had high levels of peripheral IFN-α. Furthermore, ICs formed by MDA5 and anti-MDA5, either from DM or SLE, are potent IFN-α inducers. The induction of IFN-α is more prominent in the DM-derived anti-MDA5 ICs, which probably reflects a higher affinity effect. As expected, RNA binding is required and interaction with TLR7 is evident in anti-MDA5 ICs. The origin and function of binding RNA will be another intriguing question that deserves further study. Nevertheless, our data implicated a possible vicious loop in the pathogenesis of anti-MDA5^+^ DM, i.e., trigger-activated (viral) type I IFN pathway and subsequent adaptive immune response (including the generation of anti-MDA5**^+^** autoantibody). In the meantime, anti-MDA5 and its ICs further enhance IFN-α production. A limitation of this study is that the current analysis was performed without including DM-specific antibody-negative DM patients. Moreover, the anti-MDA5 antibody detection in DM patients enrolled in this study was not performed using the gold standard anti-MDA5 antibody IP assay, but with EUROLINE Autoimmune Inflammatory Myopathies 16 Ag assays.

Our data provided additional evidence to understand the anti-MDA5 autoantibody-mediated, type I IFN-centered mechanism of this disorder. It was made more promising by tackling the IFN pathway [such as through Janus kinase (JAK) inhibitors] as a new treatment approach for anti-MDA5^+^ DM and associated diseases (19, 20).

## Data Availability Statement

The original contributions presented in the study are publicly available. This data can be found here: https://www.jianguoyun.com/p/DchBrVwQksbYCRiN84IE.

## Ethics Statement

The studies involving human participants were reviewed and approved by the Ethics Committee of Renji Hospital, Shanghai Jiao Tong University School of Medicine (ethics no. IRB# 2017-041). The patients/participants provided written informed consent to participate in this study.

## Author Contributions

All authors listed have made a substantial, direct, and intellectual contribution to the work and approved it for publication.

## Funding

This study was supported by the Clinical Research Plan of SHDC (no. SHDC2020CR1015B) and the National Precision medicine research program (no. 2017YFC0907602).

## Conflict of Interest

The authors declare that the research was conducted in the absence of any commercial or financial relationships that could be construed as a potential conflict of interest.

## Publisher’s Note

All claims expressed in this article are solely those of the authors and do not necessarily represent those of their affiliated organizations, or those of the publisher, the editors and the reviewers. Any product that may be evaluated in this article, or claim that may be made by its manufacturer, is not guaranteed or endorsed by the publisher.

## References

[1] Sato S Hoshino K Satoh T Fujita T Kawakami Y Fujita T. RNA Helicase Encoded by Melanoma Differentiation-Associated Gene 5 Is a Major Autoantigen in Patients With Clinically Amyopathic Dermatomyositis: Association With Rapidly Progressive Interstitial Lung Disease. Arthritis Rheum (2009) 60(7):2193–200. doi: 10.1002/art.24621 19565506

[2] Wu WL Guo L Fu YK Wang KW Zhang DT Xu WW. Interstitial Lung Disease in Anti-MDA5 Positive Dermatomyositis. Clin Rev Allergy Immunol (2021) 60(2):293–304. doi: 10.1007/s12016-020-08822-5 33405101

[3] Brisse M Ly H. Comparative Structure and Function Analysis of the RIG-I-Like Receptors: RIG-I and MDA5. Front Immunol (2019) 10:1586. doi: 10.3389/fimmu.2019.01586 31379819 PMC6652118

[4] Paik JJ Livia Casciola-Rosen L Shin JY Albayda J Tiniakou E Leung DG. Study of Tofacitinib in Refractory Dermatomyositis: An Open-Label Pilot Study of Ten Patients. Arthritis Rheum (2021) 73(5):858–65. doi: 10.1002/art.41602 PMC8084900 33258553

[5] Rönnblom L Alm GV Eloranta M-L. The Type I Interferon System in the Development of Lupus. Semin Immunol (2011) 23:113–21. doi: 10.1016/j.smim.2011.01.009 21292501

[6] Enevold C Kjær L Nielsen CH Voss A Jacobsen RS Hermansen MLF. Genetic Polymorphisms of dsRNA Ligating Pattern Recognition Receptors TLR3, MDA5, and RIG-I. Association With Systemic Lupus Erythematosus and Clinical Phenotypes. Rheumatol Int (2014) 34(10):1401–8. doi: 10.1007/s00296-014-3012-4 24719229

[7] Graham DSC David L Morris DL Bhangale TR Criswell LA Syvänen AC. Association of NCF2, IKZF1, IRF8, IFIH1, and TYK2 With Systemic Lupus Erythematosus. PLoS Genet (2011) 7(10):e1002341. doi: 10.1371/journal.pgen.1002341 22046141 PMC3203198

[8] Horai Y Koga T Fujikawa K Takatani A Nishino A Nakashima Y. Serum Interferon-α Is a Useful Biomarker in Patients With Anti-Melanoma Differentiation-Associated Gene 5 (MDA5) Antibody-Positive Dermatomyositis. Mod Rheumatol (2015) 25(1):85–9. doi: 10.3109/14397595.2014.900843 24716595

[9] Zhang SH Zhao Y Xie QB Jiang Y Wu YK Yan B. Aberrant Activation of the Type I Interferon System may Contribute to the Pathogenesis of Anti-Melanoma Differentiation-Associated Gene 5 Dermatomyositis. Br J Dermatol (2019) 180(5):1090–8. doi: 10.1111/bjd.16917 29947075

[10] Dias Junior AG Sampaio NG Rehwinkel J. A Balancing Act: MDA5 in Antiviral Immunity and Autoinflammation. Trends Microbiol (2019) 27(1):75–85. doi: 10.1016/j.tim.2018.08.007 30201512 PMC6319154

[11] Muro Y Sugiura K Hoshino K Akiyama M Tamakoshi K. Epidemiologic Study of Clinically Amyopathic Dermatomyositis and Anti-Melanoma Differentiation-Associated Gene 5 Antibodies in Central Japan. Arthritis Res Ther (2011) 13(6):R214. doi: 10.1186/ar3547 22192091 PMC3334667

[12] Mammen AL Allenbach Y Stenzel W Benveniste O ENMC 239th Workshop Study Group. 239th ENMC International Workshop: Classification of Dermatomyositis, Amsterdam, the Netherlands, 14-16 December 2018. Neuromuscul Disord (2020) 30(1):70–92. doi: 10.1016/j.nmd.2019.10.005 31791867

[13] Wang H Li T Chen S Gu Y Ye S. Neutrophil Extracellular Trap Mitochondrial DNA and Its Autoantibody in Systemic Lupus Erythematosus and a Proof-Of-Concept Trial of Metformin. Arthritis Rheumatol (2015) 67(12):3190–200. doi: 10.1002/art.39296 26245802

[14] Feng XB Wu H Grossman JM Hanvivadhanakul P FitzGerald JD Park GS. Association of Increased Interferon-Inducible Gene Expression With Disease Activity and Lupus Nephritis in Patients With Systemic Lupus Erythematosus. Arthritis Rheum (2006) 54(9):2951–62. doi: 10.1002/art.22044 16947629

[15] Sato S Murakami A Kuwajima A Takehara K Mimori T Kawakami A. Clinical Utility of an Enzyme-Linked Immunosorbent Assay for Detecting Anti-Melanoma Differentiation-Associated Gene 5 Autoantibodies. PLoS One (2016) 11(4):e0154285. doi: 10.1371/journal.pone.0154285 27115353 PMC4846082

[16] Wu WL Xu WW Sun WJ Zhang DT Zhao JF Luo Q. Forced Vital Capacity Predicts the Survival of Interstitial Lung Disease in Anti-MDA5 Positive Dermatomyositis: A Multi-Centre Cohort Study. Rheumatol (Oxford) (2021) keab305. doi: 10.1093/rheumatology/keab305 33764398

[17] Eloranta M-L Lövgren T Finke D Mathsson L Rönnelid J Kastner B. Regulation of the Interferon-Alpha Production Induced by RNA-Containing Immune Complexes in Plasmacytoid Dendritic Cells. Arthritis Rheum (2009) 60(8):2418–27. doi: 10.1002/art.24686 19644885

[18] Mold C Clos TWD. C-Reactive Protein Inhibits Plasmacytoid Dendritic Cell Interferon Responses to Autoantibody Immune Complexes. Arthritis Rheum (2013) 65(7):1891–901. doi: 10.1002/art.37968 PMC3701728 23576062

[19] Chen ZW Wang XD Ye S. Tofacitinib in Amyopathic Dermatomyositis-Associated Interstitial Lung Disease. N Engl J Med (2019) 381(3):291–3. doi: 10.1056/NEJMc1900045 31314977

[20] Kurasawa K Arai S Namiki Y Tanaka A Takamura Y Owada T. Tofacitinib for Refractory Interstitial Lung Diseases in Anti-Melanoma Differentiation-Associated 5 Gene Antibody-Positive Dermatomyositis. Rheumatol (Oxford) (2018) 57(12):2114–9. doi: 10.1093/rheumatology/key188 30060040

